# Impact of Vaginal Dilator Use and 68 Gy EQD2_(α/β=3)_ Dose Constraint on Vaginal Complications in External Beam Irradiation Followed by Brachytherapy in Post-Operative Endometrial Cancer

**DOI:** 10.3390/jpm14080838

**Published:** 2024-08-08

**Authors:** Faegheh Noorian, Rosa Abellana, Yaowen Zhang, Antonio Herreros, Valentina Lancellotta, Luca Tagliaferri, Sebastià Sabater, Aureli Torne, Eduard Agusti-Camprubi, Angeles Rovirosa

**Affiliations:** 1 Fonaments Clínics Department, Universitat de Barcelona, 08036 Barcelona, Spain; faegheh.noorian@gmail.com (F.N.); rabellana@ub.edu (R.A.); herreros@clinic.cat (A.H.); 2 Radiation Oncology Department, Hospital Clinic, Universitat de Barcelona, 08036 Barcelona, Spain; 3 Cancer Center, Henan Provincial People’s Hospital, No.7 Weiwu Road, Zhengzhou 450003, China; 250522926@qq.com; 4 Dipartimento di Diagnostica per Immagini, Radioterapia Oncologica ed Ematologia, Policlinic Universitario Gemelli, 00168 Rome, Italy; valentina.lancellotta@policlinicogemelli.it (V.L.); luca.tagliaferri@policlinicogemelli.it (L.T.); 5 Radiation Oncology Department, Hospital General Universitario de Albacete, 02006 Albacete, Spain; ssabaterm@gmail.com; 6 Gynaecological Cancer Unit, Hospital Clinic, Universitat de Barcelona, 08036 Barcelona, Spain; atorne@clinic.cat; 7 Radiological Protection Department, Hospital Clinic, Universitat de Barcelona, 08036 Barcelona, Spain; agusti@clinic.cat; 8 Institut d’Investigació Biomèdica Agustí Pi i Sunyer (IDIBAPS), 08036 Barcelona, Spain

**Keywords:** brachytherapy schedules, complications, endometrial cancer

## Abstract

Background: This study evaluated the clinical outcomes of applying a 68 Gy EQD2_(α/β=3)_ dose constraint to the most exposed 2 cm^3^ area of the vagina in post-operative endometrial cancer patients treated with vaginal-cuff brachytherapy after external beam irradiation and the impact of vaginal dilator use on late vaginal complications. Material and methods: We analyzed 131 patients treated with vaginal-cuff brachytherapy after external beam irradiation. Group-1 (65 patients) received one fraction of 7 Gy, and Group-2 (66 patients) received one fraction of between 5.5 and 7.0 Gy after applying a 68 Gy EQD2_(α/β=3)_ dose constraint. Vaginal-cuff relapse, late toxicity, clinical target volume, vaginal dilator use, D90, and EQD2_(α/β=3)_ at 2 cm^3^ of the most exposed part of the clinical target volume were evaluated. Descriptive analysis, the chi-squared test, Student’s *t*-test, and the Cox proportional and Kaplan–Meier models were used for the statistical analysis. Results: With a median follow-up of 60 months, the vaginal-cuff relapse rate was 1/131 (0.8%). Late vaginal complications appeared in 36/65 (55.4%) Group-1 patients and 17/66 (25.8%) Group-2 patients (*p* = 0.003). Multivariate analysis showed that belonging to Group-1 and vaginal dilator use of <9 months were independent prognostic factors of late vaginal complications with hazard ratios of 1.99 (*p* = 0.021) and 3.07 (*p* = 0.010), respectively. Conclusions: A 68 Gy EQD2_(α/β=3)_ constraint at 2 cm^3^ of clinical target volume and vaginal dilator use of ≥9 months were independent prognostic factors, having protective effects on late vaginal complications.

## 1. Introduction

Endometrial cancer is the sixth most common malignant disorder worldwide and the most frequent gynecological cancer. The treatment of endometrial cancer involves total hysterectomy and bilateral salpingo-oophorectomy with or without lymph node assessment. The indication for adjuvant radiation therapy is based on stage, tumor type, and the presence of risk factors including molecular factors. Most recurrences (65–85%) are diagnosed within 3 years of primary treatment, and 40–75% of recurrences are in the vagina [1,2,3].

According to the data from randomized trials, no adjuvant treatment is recommended in patients with low-risk FIGO Stage IA grade 1 or 2 endometrial carcinoma. Adjuvant vaginal-cuff brachytherapy (VCB) is recommended to decrease vaginal recurrence in patients with intermediate or high-intermediate risk. For high-intermediate risk patients, external beam radiation therapy (EBRT) can be considered in cases of substantial lymphovascular space invasion (LVSI), p53 mutation, and for stage II endometrial cancer. Adjuvant brachytherapy can be recommended to reduce vaginal recurrence. Adjuvant chemotherapy can be considered, especially in high-grade endometrial cancer and/or cases with substantial LVSI. In high-risk patients, EBRT with concurrent and adjuvant chemotherapy or, alternatively, sequential chemotherapy and radiotherapy is recommended. Nevertheless, the administration of VCB varies among centers and some recent studies have shown the improvements in local vaginal control with the use of VCB + EBRT in the advanced stages [3,4,5,6,7]. 

The most common EBRT doses range from 45 to 50.4 Gy delivered in 25 to 28 fractions over 5 to 6 weeks. Since 2019, the gold standard has been the intensity-modulated radiation therapy and volumetric-modulated arc therapy (VMAT) techniques [8]. The American Brachytherapy Society (ABS) has reported a wide variation in VCB dose schedules, including 22 regimens used as a boost after EBRT [9].

The organs at risk (OAR) in pelvic radiation therapy include the bladder, rectum, and vagina. The incidence of late rectal and bladder complications is more closely associated with EBRT, while VCB is more often related to the development of late vaginal complications (LVCs), which mostly occur after combined treatment with VCB and EBRT. A commonly observed LVC is Grade-1 (G1) and Grade-2 (G2) radiation therapy (RT)-induced vaginal stenosis (VS), defined as the abnormal tightening and shortening of the vagina due to the formation of adherences and fibrosis. It is well recognized that RT-induced advanced VS may have a negative impact on patient well-being, especially in relation to sexual dysfunction and dyspareunia and limiting physical examination in post-treatment follow-up. The main factors associated with the development of LVCs, mainly stenosis, are vaginal surface doses, cylinder diameter, high dose per fraction, active source length, and vaginal dilator (VD) use. However, there is no consensus on the ideal length of time of VD use and dose-fractionation schedules [10,11,12,13,14,15].

The Groupe Européen de Curiethérapie of the European Society for Radiotherapy and Oncology (GEC-ESTRO) and other groups recommend EQD2_(α/β=3)_ at the most exposed 2 cm^3^ of normal tissue as a limit for the bladder, rectum, and sigmoid. Hence, several years ago, we hypothesized that EQD2_(α/β=3)_ at the most exposed 2 cm^3^ of the vagina (which we also consider as an OAR) could be a predictor of vaginal toxicity. On analyzing the correlation of dose with post-operative toxicity in patients treated with high-dose rate (HDR) VCB, we found that a dose constraint of 68 Gy EQD2_(α/β=3)_ at 2 cm^3^ was associated with vaginal toxicity ≥ G2. Therefore, this dose limit has been applied for reducing vaginal toxicity since 2017 [13,16,17,18].

Prior to 2017, patients would receive a single dose of 7 Gy after EBRT. However, from 2017 to 2022, we prospectively applied the restriction of the EQD2_(α/β=3)_ to 68 Gy at 2 cm^3^ of the most exposed vaginal clinical target volume (CTV) in 79 patients treated with EBRT + VCB. Preliminary results showed a reduction in LVCs in patients when the constraint was applied [13].

In a recent study on exclusive VCB using 7.5 Gy × 2 fractions in 110 patients, the use of the 68 Gy constraint did not seem useful (only four patients received more than this constraint) [19]. Therefore, the aim of this study was to retrospectively compare vaginal control and LVCs in a group of patients receiving one dose of 7 Gy of VCB after EBRT, with a second group receiving a 68 Gy EQD2_(α/β=3)_ dose constraint to the most exposed 2 cm^3^ of the vagina. In addition, we evaluated the impact of vaginal dilator use greater or less than 9 months on the development of vaginal complications. This is the first study evaluating these data using 3D treatment planning in VCB.

## 2. Materials and Methods

The present study was approved by the Institutional Ethical Review Board of our center (HCB 2022/0379), and patient consent for study participation was obtained. We retrospectively analyzed 159 post-operative endometrial cancer (PEC) patients treated with EBRT followed by VCB from 2014 to 2022. Among these 159 patients, 28 (14 patients from each group) were excluded due to a lack of at least 12 months of follow-up or related assessments, and thus a total of 131 patients were analyzed.

**Figure 1:**
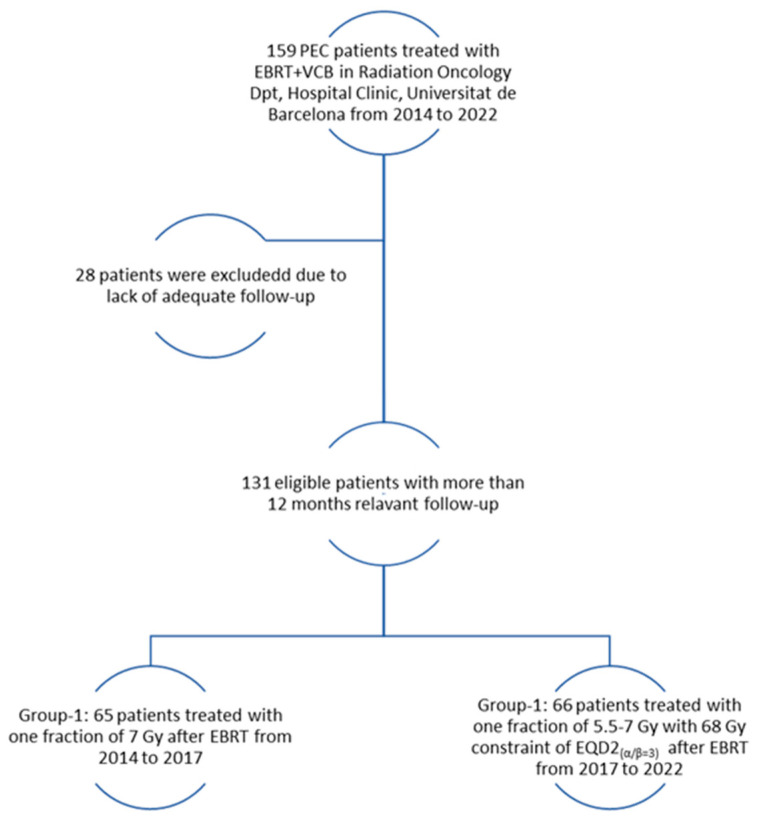
Inclusion and exclusion flowchart.

From 2014 to 2017, 65 patients were treated with EBRT + VCB using 1 fraction of 7 Gy after EBRT and underwent adequate follow-up (Group-1). Subsequently, from 2017 to 2022, 66 patients were treated with EBRT+ VCB, receiving one fraction of 5.5 to 7 Gy while ensuring a constraint of EQD2_(α/β=3)_ < 68 Gy at 2 cm^3^ of the most exposed part of the vaginal CTV before receiving adequate follow-up (Group-2). Figure 1 shows the patient selection process and the treatment received.

Following the diagnosis of endometrial cancer, patients underwent imaging workup studies, including magnetic resonance imaging, positron emission tomography, computerized tomography, and/or ultrasonography. Subsequently, all patients underwent surgery involving the following surgical approaches: laparoscopic-assisted vaginal hysterectomy and bilateral salpingo-oophorectomy (LAVH-BSO) with pelvic ± para-aortic lymphadenectomy in 50 (38.2%) patients, LAVH-BSO with pelvic ± para-aortic lymphadenectomy in 30 (22.9%) patients, vaginal hysterectomy in 5 (3.8%) patients, abdominal hysterectomy in 3 (2.3%) patients, and an omentectomy in 13 (9.9%) patients. Thirty (22.9%) patients were treated using other methods, such as a LAVH-BSO by robotic surgery.

Following pathological analysis, all patients received EBRT + VCB, and 4–6 cycles of chemotherapy (carboplatin/paclitaxel) were administered to 54 patients (41.2%), according to their general status, age, and comorbidities. 

EBRT was delivered with 6 or 18 MV photons to 127/131 (96.9%) patients after 3D treatment planning, while 4 (3.1%) patients received EBRT by VMAT. The delineation of the CTV and the planning target volume were performed following the Radiation Therapy Oncology Group (RTOG) protocols (20). The dose per fractionation ranged from 1.8 to 2 Gy per day, administered as 5 fractions per week. When positive lymph nodes were present, a dose of up to 65 Gy was used.

After finishing EBRT, VCB was performed. Applicators were placed in the operating room, where the patients were first examined, to confirm the type and diameter of the applicator to use. A colpostat is preferred in patients with a small introitus and wide vagina, while a vaginal cylinder is commonly used for uniform and adequate anatomy. 

The Oncentra Brachy planning system (Elekta^®^, Nucletron BV, Veenendaal, The Netherlands) was used for treatment planning. The vaginal CTV was delineated at 2.5 cm along the first cylinder. The VCB dose was prescribed at 0.5 cm from the applicator surface with optimization to points. A 90% isodose was considered to cover all the CTV of the vagina. The brachytherapy planning technique has been described elsewhere (13).

Follow-up was carried out 15 days after brachytherapy and then every 3–4 months during the first 2 years and every 6 months thereafter up to 5 years. The patients were evaluated in terms of recurrence and side effects by clinical and gynecological examination and imaging studies. All patients were visited by the same radiation oncologist during and after EBRT, before VCB, and during follow-up. All patients were advised to use VDs, adapted to their vaginal size. The follow-up period for the analysis was defined from the date of VCB until the last visit of the patient, and the LVC-free time was defined as the time between VCB and the appearance of LVCs.

We analyzed these two VCB dose fractionation groups in relation to various factors, including vaginal-cuff relapse (VCR), late toxicities in the vagina, rectum, and bladder, CTV, the use of VDs ≥ 9 months versus no usage or <9 months, D90, EQD2_(α/β=3)_ at 2 cm^3^ of the most exposed part of CTV of VCB, with the corresponding overall value representing the cumulative dose of VBT + EBRT.

Late toxicity of the rectum and bladder was assessed using the RTOG scores, and LVCs were evaluated with the objective criteria of LENT-SOMA [20,21].

Statistical analysis: Categorical variables were expressed using frequencies and percentages, while continuous data were described using mean and standard deviation (SD) or median and interquartile range. The homogeneity study between dose regimen groups was performed using the chi-squared test or Fisher’s Exact Test for the categorical variables, or Student’s *t*-test in the case of continuous variables. The mean, median, or proportion differences between dose regimen groups were estimated with a 95% confidence interval (CI). 

We identified potential prognostic factors by univariate analysis. Subsequently, variables that showed significance in the univariate analysis were included in the multivariable Cox proportional hazards regression model. This model adjusts for the simultaneous influence of multiple factors on the hazard of developing an LVC over time, and hazard ratios (HR) were estimated with a 95% CI.

Due to varying follow-up times, we adopted a Kaplan–Meier survival analysis approach to effectively capture the time-to-event outcome of interest, which, in this case, was the occurrence of LVCs. Additionally, we employed the Kaplan–Meier estimator to graphically depict the probability of remaining free from LVCs at each month. The effect of the dose regimen and other prognostic factors on the probability of LVC-free time was investigated using the Cox proportional hazards model. This model is particularly suited for survival data as it allows for an assessment of the relative hazards (risks) of developing LVCs between groups, while accounting for varying follow-up durations and censoring. The analyses were performed using R software version 4.2.2 package (R project for statistical computing; Vienna, Austria).

## 3. Results

We analyzed 131 eligible patients with a median follow-up of 60 months (15–60) in Group-1 and 46 months (14–60) in Group-2. Among the entire series, 16 patients died. Of these, one patient in each group died as a consequence of endometrial cancer within 14–15 months of follow-up. 

Table 1 shows the comparison of prognostic factors of local recurrence between the two study groups, which were homogenous except for histologic grade and focal LVSI.

In the present study, VCR was observed in only one patient (0.8%) in Group-1, who had received 7 Gy and died 7 months after relapse due to causes unrelated to cancer. In 3/66 (4.5%) patients in Group-2, VCR occurred in the middle or outer third of the vagina, outside the field of brachytherapy. Two of these patients received 6.2 Gy and one received 6.5 Gy. These three patients remained alive after treatment for VCR.

Table 2 shows the dosimetry parameters and characteristics of the brachytherapy administered. The median EQD2_(α/β=3)_ of EBRT was 45 Gy (40;50) in both groups. The mean VCB EQD2_(α/β=3)_ at 2 cm^3^ of CTV was 27.4 Gy in Group-1 and 22.9 Gy in Group-2 (*p* < 0.001). All the Group-1 patients received more than 68 Gy overall EQD2_(α/β=3)_, and all the patients in Group-2 received less than this dose.

The dose per fraction in Group-2 was as follows: 5.5 Gy 1/66 (1.5%), 5.7 Gy 1/66 (1.5%), 6.0 Gy 3/66 (4.5%), 6.2 Gy 13/66 (19.7%), 6.5 Gy 37/66 (56.1%), 6.75 Gy 6/66 (9.1%), and 7.0 Gy 5/66(7.6%). The mean dose per fraction in Group-2 was 6.5 Gy (SD 0.3) with a median of 6.5 Gy (5.5;7).

In Group-1, 2/65 (3.1%) patients presented late rectal complications; one G1 and one G2. In Group-2, 2/66 (3.0%) patients developed G1 late rectal complications (*p* = 1.0). Late bladder complications were observed in 2/66 (3.0%) in Group-2; one G1 and one G2 (*p* = 0.5).

Considering LVCs, 7/65 (10.8%) Group-1 patients presented Grade-1 (G1-LVC) as small adhesions, while 11/65 (16.9%) presented adhesions or simultaneous small dog ear (a retraction of <1 cm located in the vaginal cuff corners on vaginal digital examination) and telangiectasia, and 3/65 (4.6%) presented a small dog ear alone while 8/65 (12.3%) presented telangiectasia. Grade-2 LVCs (G2-LVCs) appeared as vaginal shortening or cleisis in 3/65 (4.6%) patients and bleeding adhesion in 4/65 (6.2%) patients. Group-2 presented G1-LVCs as small adhesions in 5/66 (7.6%) patients, small dog ears in 2/66 (3.0%), telangiectasia in 3/66 (4.5%), adhesions or small dog ears and telangiectasia at the same time in 3/66 (4.5%) patients, and vaginal shortening of less than one-third in 1/66 (1.5%). G2-LVCs were observed in 3/66 (4.5%) patients, all in the form of vaginal shortening of between 1/3 and 2/3. 

In the whole series, 112/131 (85.5%) patients received more than 6.5 Gy; among these, 39/112 (34.8%) patients presented G1-LVCs and 10/112 (8.9%) G2-LVCs. Up to 19 of the 131 (14.5%) patients received < 6.5 Gy per fraction, with only 4/19 (21.1%) presenting G1-LVCs, and no G2-LVCs were observed (*p*-value = 0.06). Among the Group-2 patients who received more than 6.5 Gy per fraction, 10/47 (21.3%) presented G1-LVCs and 3/47 (6.4%) patients had G2-LVCs (*p*-value = 0.78). 

Table 3 shows the univariate analysis of possible prognostic factors associated with the appearance of LVCs. The median time to LVC appearance was 12.9 (0.4; 60.0) months in the whole series; however, the mean time to G2-LVC development was longer in Group-2, with 22.1 (SD 13.6) months in Group-1 and 39.2 (SD4.5) in Group-2 (*p*-value < 0.01). Table 3 shows that, in the present study, VD use of ≥9 months, a lower VCB dose per fraction, and consequently belonging to treatment Group-2 demonstrated a protective effect against the onset of LVCs over time.

Table 4 shows the results of the multivariable analysis of prognostic factors of time to the appearance of the LVCs. This analysis revealed that VD use of <9 months was associated with a HR of 3.07 (95% CI 1.30, 7.23; *p* = 0.010), indicating a significant three times higher risk of developing LVCs compared to VD use ≥ 9 months. Furthermore, the multivariate model identified group assignment as an independent prognostic factor, with Group-1 demonstrating a HR of 1.99 (95% CI 1.11, 3.55; *p* = 0.021) compared to Group-2. 

**Figure 2:**
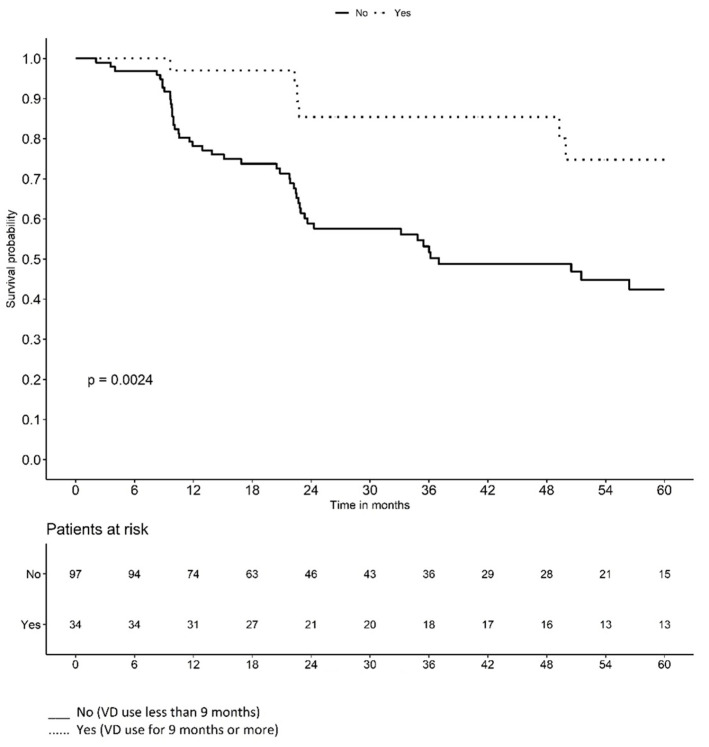
Probability of late vaginal complication-free time according to vaginal dilator use.

**Figure 3:**
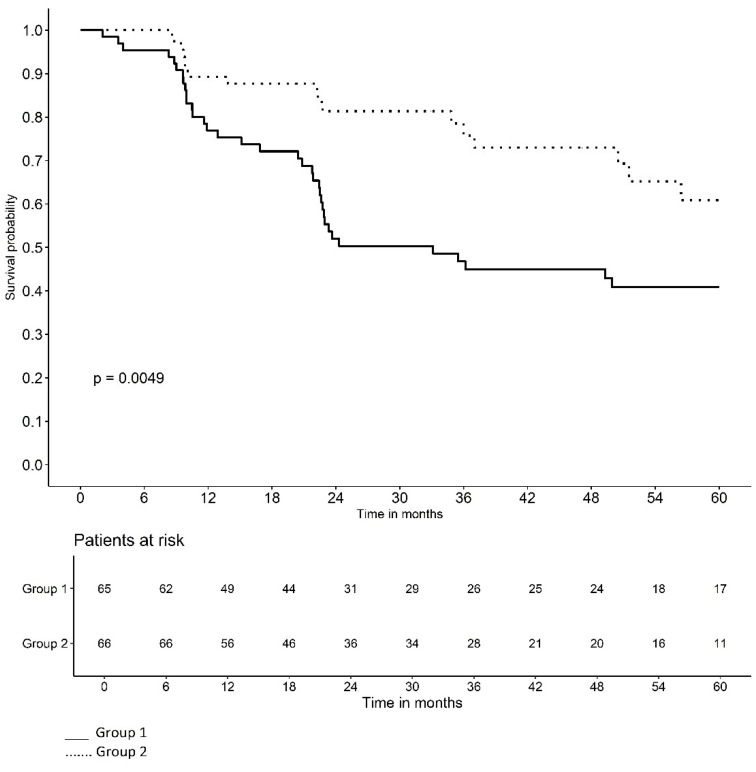
Probability of late vaginal complication-free time according to treatment group.

Figure 2 and Figure 3 illustrate the Kaplan–Meier survival curves for the probability of LVC-free time based on the duration of VD use and treatment group. At the beginning of the study, the probability of remaining LVC-free was high in all patients. However, over time, the probability of remaining LVC-free decreased with the appearance of complications. The curves for each group slope downward at different rates, reflecting the monthly probability of LVC-free survival in each group. Note that the curve for VD use of <9 months and the curve for Group-1 fell more steeply.

## 4. Discussion

The present study analyzed the prognostic factors for LVCs in patients with PEC receiving EBRT + VBT. The aim of the study was not to establish the role of VBT in advanced stages of PEC, since there is no agreement between centers and some guidelines and authors have reported advantages in combining VBT with EBRT to enhance local vaginal control as in the present series [1,2,3,4].

The rate of VCR reported in the literature varies from 1.5% to 7% [2,10,14,19]. In the present study, the VCR rate was 0.8% in the entire series, with one patient in Group-1. Due to the low VCR rate, the difference between the two groups was not statistically significant.

The data available regarding the incidence and grading of LVCs are based on retrospective evidence and small cohorts, with a wide variation in the LVC measurement techniques and fractionation schedules as shown in the literature, and the reported rates of VS ranging from 1.3% to 88.0% based on the CTCAE or LENT-SOMA scores. In our previous studies, the incidence of G1-LVCs varied from 13% to 30%, while that of G2-LVCs was from 13% to 15% in patients receiving VCB after EBRT, and the incidence of VS was 10%, being very similar to our current study [11,21,22]. 

In our previous retrospective studies, univariate analysis showed that in patients > 55 years of age, an EQD2_(α/β=3)_ of more than 68 Gy at the most exposed 2 cm^3^ of the CTV and the use of VD for < 9 months were associated with LVCs and, in multivariate analysis, VD use of ≥9 months was an independent prognostic factor for G2-LVCs in patients receiving VCB ± EBRT [23]. However, in a recent study by our group on exclusive VCB in PEC patients, the application of a 68 Gy EQD2_(α/β=3)_ constraint in exclusive VCB did not seem necessary, probably due to the lower VCB dose received by the group [19].

In the group receiving 7 Gy after EBRT in the present study, there was a higher incidence of complications, and all these patients received > 68 Gy EQD2_(α/β=3)_ to the most exposed 2 cm^3^ of the vagina. The present study highlights that a 68 Gy EQD2_(α/β=3)_ vaginal dose constraint and VD use of ≥9 months in PEC patients treated with EBRT + VCB are independent prognostic factors of LVCs. 

It is assumed that a higher volume of vagina treated is associated with an increased risk of VS. In the EMBRACE I study on cervical cancer, the 24-month actuarial estimate for symptomatic VS was 21%, and the vaginal tumor invasion into the vagina was reported to be a considerable risk factor [24]. Nevertheless, the EMBRACE I study included cervical tumors treated with a curative aim and with vaginal invasion in some of the cases, and thus higher doses were administered to the vagina. In the present series of PEC treatment, all the patients were treated with same vaginal CTV longitude and the mean CTV was similar in both groups, and therefore the results were not affected. Moreover, in the univariate analysis, there was no correlation between the CTV and LVCs. Hintz et al. suggested that high vaginal toxicity observed in their study may also have been due to the irradiation of the lower third of the vagina, which is more sensitive to radiotherapy, but this was not the case in the present study [25]. Glatzer et al. reported that 71% of the experts in their study administered the treatment to the upper 3 cm of the vagina, which could be considered as a reference in vaginal CTV delineation for reducing vaginal complications associated with higher vaginal-treated volumes [6].

The use of an applicator diameter of <2.5 cm has been associated with a higher incidence of G1–2 VS [26]. In the present study, univariate analysis did not show a correlation between the applicator diameter and the LVCs (Table 3). Nonetheless, the number of patients treated with an applicator diameter of 2.5 cm or less was too low to establish conclusions (13/131). Moreover, it should be noted that the use of the constraint of 68 Gy of the applicator diameter rules out an association between the applicator diameter and the LVCs.

The EBRT dose used is commonly 45/0–50.4 Gy in 25–28 fractions over 5–6 weeks. Radiation doses to vaginal surface > 80 Gy have been associated with a 10% to 15% increased risk of G2-LVCs, including VS. Maintaining an EBRT dose of 45 Gy/25 fractions and decreasing the brachytherapy dose to the vagina reduces the risk of VS [27]. In our study, it is unlikely that the EBRT dose affected the comparison of the outcomes of the two groups, considering that there was no significant difference between the mean EBRT dose between the two groups. The addition of a VCB boost to EBRT should generally result in a vaginal surface low-dose rate (EBRT and brachytherapy) equivalent to 65–70 Gy. The HDR VCB fractionation recommended by the ABS is 5–6 Gy in three fractions or 6 Gy in two fractions prescribed to the surface after 45 Gy or 50.4 Gy fractions of EBRT [2,14,24,28]. 

This study has shown that higher doses to the most exposed 2 cm^3^ of the CTV are associated with an increase in complications as suggested in previous studies by our group. Although G1 complications have little clinical impact, the mentioned constraint would likely reduce G2-LVCs. Thus, the use of this constraint seems to have a protective effect against G1- and G2-LVCs, especially in G2 vaginal shortening. Nonetheless, studies with more participants are necessary to confirm these findings.

The mean dose per fraction was higher in patients showing G1- or G2-LVCs at 6.9 Gy (SD 0.3) compared to 6.6 Gy (SD 0.3) in those without LVCs. Despite the lack of statistical differences in the incidence of LVCs between patients receiving ≥ or <6.5 Gy (*p* = 0.10), only 4 out of 19 (21.1%) patients showed G1-LVCs, and no G2-LVCs were observed among 19/131 (14.5%) patients who received < 6.5 Gy per fraction. Considering the LVCs and the EQD2, we can hypothesize that an exclusive fraction of between 6.0 Gy and 6.5 Gy could be enough to reduce complications in these patients. 

Following vaginal or pelvic radiotherapy VD use is recommended to prevent VS. However, the time between the development of VS and the optimal length of time of VD use remain unknown. Stahl et al. proposed that the risk of VS persists beyond 1 year after brachytherapy. VD compliance beyond 1 year may mitigate this risk. The Delphi method encourages women to begin using a VD four weeks after completing RT treatment, 1–3 min, 2–3 times per week, and for 9 to 12 months. The Brazilian consensus recommended that VD use last at least 5–10 min, 2–3 times per week for an indefinite time [12,29,30,31,32,33,34]. After our previous multivariate analysis showing that VD use > 9 months reduced G2-LVCs in patients who complied with usage 2–3 days a week, our patients were encouraged to use VD daily during the 5-year follow-up. In the current study, only one patient with G1-VS and no patient with G2-VS reported VD use of ≥9 months. This might confirm the protective role of VD use of ≥9 months in the appearance and severity of LVCs and VS. The present analysis confirmed the results of our previous retrospective study. Moreover, the EMBRACE II study reported the benefits of VD use in cervical cancer [35].

Univariate analysis of possible prognostic factors of possible LVC-free time, comparing patients with and without LVCs, showed that belonging to treatment Group-2 and VD use ≥ 9 months have protective effects against the onset of LVCs over time. Considering the fact that all the patients in Group-2 received < 68 Gy overall EQD2_(α/β=3)_ at the most exposed 2 cm^3^ of vaginal CTV, it could be concluded that a 68 Gy constraint has a positive effect on LVC-free time as well as LVC severity. 

Multivariate analysis revealed that belonging to the treatment group and VD use are independent prognostic factors. Patients in Group-1 had nearly a 2-fold greater risk of developing LVCs compared to Group-2. By analyzing the interaction between VD use ≥ 9 months and treatment group, it was observed that, depending on the dose group, there were no differences in the effect of VD use ≥ 9 months, showing that VD use and dose group are independent prognostic factors with an impact on LVCs (Table 4). This reinforces the importance of considering both the treatment regimen and the duration of VD use in the clinical management and risk assessment of LVCs. 

The Kaplan–Meier survival curves for the probability of LVC-free time based on the duration of VD use and treatment group illustrate that the curve for VD use < 9 months and the curve for Group-1 fell more steeply, indicating a lower monthly probability of remaining LVC-free (Figure 2 and Figure 3). In other words, belonging to Group-2 and VD use ≥ 9 months have a protective effect on the time of LVC development.

Considering the present results, the effect of VD use remains consistent in PEC treated with EBRT + VCB regardless of the dose received and treatment group.

The limitations of this study were the number of patients included with a low number of VCR, LVC, and VS cases available for analysis. Nevertheless, the results are promising enough to initiate a prospective study to further analyze this topic. Additionally, future studies should consider quality of life metrics, sexual function, and variations in patterns of VD use, including the number of days and other associated medical treatments.

## 5. Conclusions

Considering that all the patients in Group-2 received less than 68 Gy EQD2_(α/β=3)_ at 2 cm^3^ of CTV and had a higher probability of LVC-free time, it can be concluded that this constraint could prevent the development of LVCs while maintaining the same vaginal control. Since the mean dose per fraction in Group-2 was 6.5 Gy, it could be hypothesized that one fraction of 6.0–6.5 Gy is an effective VCB boost after EBRT in patients with PEC. Moreover, VD use ≥ 9 months has an independent protective effect on the incidence of LVCs. In order to confirm these results, studies with a larger number of participants are needed.

## Figures and Tables

**Table 1:** The clinical characteristics of the entire sample of patients and by study group.

	All	Group-1	Group-2	*p*-Value	N
	N = 131	N = 65	N = 66		
Mean Age (SD)	65.4 (10.3)	64.5 (10.3)	66.4 (10.4)	0.289	131
Histologic grade (n, (%))				0.032	131
G1 + 2	85 (65.9%)	48 (73.8%)	37 (56.1%)		
G3	46 (35.1%)	17 (26.2%)	29 (43.9%)		
Myometrial invasion (n, (%))				0.542	131
<50%	48 (36.6%)	26 (40.0%)	22 (33.3%)		
≥50%	83 (63.4%)	39 (60.0%)	44 (66.7%)		
Mean Tumor size (mm) (SD)	38.4 (16.2)	35.4 (15.5)	41 (16.4)	0.064	115
Focal LVSI (n, (%))				<0.001	128
No	64 (50.0%)	44 (69.8%)	20 (30.8%)		
Yes	64 (50.0%)	19 (30.2%)	45 (69.2%)		
Pathologic types (n, (%))				0.917	131
Endometrioid	98 (74.8%)	49 (75.4%)	49 (74.2%)		
Serous	13 (9.9%)	6 (9.2%)	7 (10.6%)		
Clear cell	6 (4.6%)	2 (3.1%)	4 (6.1%)		
Mix	14 (10.7%)	8 (12.3%)	6 (9.1%)		
FIGO 2023 staging: (n, %)				0.405	131
IA	12 (9.2%)	8 (12.3%)	4 (6.1%)		
IB	38 (29%)	22 (33.8%)	16 (24.2%)		
IC	7 (5.3%)	2 (3.1%)	5 (7.6%)		
IIA	12 (9.2%)	5 (7.7%)	7 (10.6%)		
IIB	9 (6.9%)	3 (4.6%)	6 (9.1%)		
IIC	2 (1.5%)	0 (0.0%)	2 (3%)		
IIIA	7 (5.3%)	4 (6.2%)	3 (4.5%)		
IIIB	2 (1.5%)	1 (1.5%)	1 (1.5%)		
IIIC	17 (13%)	8 (12.3%)	9 (13.6%)		
IVA	15 (11.5%)	5 (7.7%)	10 (15.2%)		
IVB	3 (2.3%)	3 (4.6%)	0 (0.0%)		
IVC	7 (5.3%)	4 (6.2%)	3 (4.6%)		
Chemotherapy (n, (%))				0.646	131
No	77 (58.8%)	40 (61.5%)	37 (56.1%)		
Yes	54 (41.2%)	25 (38.5%)	29 (43.9%)		

SD: Standard deviation. LVSI: lymphovascular space invasion. N, n: number. FIGO: The International Federation of Gynecology and Obstetrics.

**Table 2:** Brachytherapy characteristics of the entire sample of patients and by study group.

	All	Group-1	Group-2	*p*-Value	N
Applicator diameter (cm)				0.219	131
2	2 (1.5%)	0 (0.0%)	2 (3.1%)		
2.5	11 (8.4%)	3 (4.6%)	8 (12.1%)		
3	19 (14.5%)	10 (15.4%)	9 (13.6%)		
3.5	99 (75.6%)	52 (80.0%)	47 (71.2%)		
Vaginal dilator use (n, (%))				0.011	131
<9 months	97 (74.0%)	55 (84.6%)	42 (63.6%)		
≥9 months	34 (26.0%)	10 (15.4%)	24 (36.4%)		
LVC (n, (%))				0.003	131
No	78 (59.5%)	29 (44.6%)	49 (74.2%)		
Grade I	43 (32.8%)	29 (44.6%)	14 (21.2%)		
Grade II	10 (7.7%)	7 (10.8%)	3 (4.6%)		
Mean CTV (SD)	8 (1.4)	8.05 (1.5)	7.92 (1.3)	0.623	117
Mean dose per fraction (SD)	6.7 (0.3)	7.0 (0.0)	6.5 (0.3)	<0.001	131
Mean D90 (SD)	7.6 (0.7)	7.9 (0.6)	7.3 (0.6)	<0.001	129
Mean Overall EQD2_(α/β=3)_ at 2 cm^3^ of CTV (SD) *	69.1 (4.2)	72.1 (3.1)	66.1 (2.6)	<0.001	131
Mean EQD2_(α/β=3)_ 2 cm^3^ of bladder (SD)	9.8 (2.5)	10.6 (2.2)	8.6 (2.5)	<0.001	110
Mean EQD2_(α/β=3)_ 2 cm^3^ of rectum (SD)	9.6 (3.0)	10.2 (2.2)	8.9 (3.7)	<0.001	110

cm: centimeters. SD: standard deviation. EQD2: Equivalent dose to a fractionation of 2 Gy per fraction. LVC: late vaginal complications; CTV: clinical target volume. D90: overall radiation dose delivered to 90% of the CTV. * Mean EQD2_(α/β=3)_ at 2 cm^3^ of CTV = Sum of EBRT and VBT.

**Table 3:** Univariate analysis of prognostic factors of the appearance of late vaginal toxicity.

	All	G0	G1–2	HR [95% CI]	*p*-Value	N
	N = 131	N = 78	N = 53			
Study group (n, (%)):					0.005	131
1	65 (49.6%)	29 (37.2%)	36 (67.9%)	2.24 [1.26;3.99]		
2	66 (50.4%)	49 (62.8%)	17 (32.1%)	Ref.		
Age (Mean, (SD)) (year):	65.4 (10.3)	66.9 (9.7)	63.3 (10.9)	0.99 [0.96;1.01]	0.275	131
Chemotherapy:					0.167	131
No	77 (58.8%)	50 (64.1%)	27 (50.9%)	Ref.		
Yes	54 (41.2%)	28 (35.9%)	26 (49.1%)	1.46 [0.85;2.50]		
Applicator diameter (cm)					0.955	131
2	2 (1.5%)	1 (1.3%)	1 (1.9%)	Ref.		
2.5	11 (8.4%)	6 (7.7%)	5 (9.4%)	0.70 [0.08;6.03]		
3	19 (14.5%)	12 (15.4%)	7 (13.2%)	0.61 [0.07;4.95]		
3.5	99 (75.6%)	59 (75.6%)	40 (75.5%)	0.73 [0.10;5.35]		
Vaginal dilator use (n, (%))					0.002	131
<9 months	97 (74.0%)	50 (64.1%)	47 (88.7%)	3.44 [1.47;8.06]		
≥9 months	34 (26.0%)	28 (35.9%)	6 (11.3%)	Ref.		
Mean dose per fraction (SD)	6.7 (0.3)	6.6 (0.3)	6.9 (0.3)	5.27 [1.73;16.1]	0.003	131

HR = Hazard Ratio.

**Table 4:** Multivariable analysis of prognostic factors of the appearance of late vaginal toxicity.

Characteristic	HR	95% CI	*p*-Value
Group			
1	1.99	1.11, 3.55	0.021
2	—	—	
Vaginal dilator use			
<9 months	3.07	1.30, 7.23	0.010
≥9 months	—	—	

HR: hazard ratio; CI: confidence interval.

## Data Availability

The data are not publicly available due to data protections regulations.
